# Supplementary material for: Examining the assumptions of heterogeneity-based management for promoting plant diversity in a disturbance-prone ecosystem
Source: PeerJ. 2019 May 3;7:e6738. doi: 10.7717/peerj.6738 (PMC6503835; doi:10.7717/peerj.6738)
Supplement: Supplemental Information 1 [file peerj-07-6738-s001.pdf]

Supplemental Tables

Table S1. The loadings of the log transformed soil cations on the first three (of eleven) Principle Components Analysis (PCA) axes for the Grid analysis. For each component the largest positive and negative loading are in bold to aid in the description of the primary axes of variation. Together the first three PC axes captured 68% of the variance in the soil variable matrix.

**Soil Cation**	**PC1**	**PC2**	**PC3**
Log P	0.09	**-0.40**	-0.35
Log Ca	**0.46**	0.13	0.04
Log Mg	0.11	**0.46**	-0.41
Log K	0.37	-0.26	-0.09
Log Na	0.13	0.39	-0.26
Log B	0.42	0.20	**0.16**
Log Fe	**-0.30**	0.28	-0.34
Log Mn	0.34	-0.28	0.05
Log Cu	0.39	0.20	-0.25
Log Zn	0.13	-0.36	**-0.51**
Log Al	-0.25	-0.16	-0.42

Table S2. The standardized β-coefficients for the OLS model of richness in the Grid analysis. The model explained 17% of variance in richness ($F_{6,121}$ = 4.13, p < 0.001).

**Explanatory Variables***	**β**	***t*-value**	***p*-value**
Soil PC1 (Comp.1)	-0.37	-4.33	< 0.001
Soil PC2 (Comp.2)	-0.19	-2.08	0.039
Soil PC3 (Comp.3)	-0.07	-0.87	0.386
Years of bison (YrsOB)	0.11	1.14	0.258
Number of burns in past 5 years (BP5Yrs)	-0.01	-0.11	0.912
Years since last burn (YrsSLB)	-0.03	-0.33	0.739

*The abbreviated name for each parameter is given in parentheses

Table S3. ANOVA table (type II) for the RDA model of composition in the Grid analysis. The model explained 16% of variance in richness ($F_{6,121} = 3.76$, $p < 0.001$). The statistics reflect the partial effect attributed to each variable after accounting for all other variables in the model.

**Explanatory Variables**	**Var**	***Df***	***F-value***	***p*-value**
Soil PC1	1.33	1	7.91	< 0.001
Soil PC2	0.66	1	3.92	< 0.001
Soil PC3	0.34	1	2.00	0.002
Years of bison	0.43	1	2.56	0.001
Number of burns in past 5 years	0.21	1	1.24	0.124
Years since last burn	0.23	1	1.38	0.041
Residuals	20.37	121		

Table S4. ANOVA table (type II) for the OLS model of richness in the Repeat analysis. The model explained 76% of variance in richness ($F_{33,206} = 19.23$, $p < 0.001$). The statistics reflect the partial effect attributed to each variable after accounting for all other variables in the model.

**Explanatory Variables***	**SS**	**DF**	***F*-value**	***p*-value**
Site ID	19061	19	21.28	< 0.001
Year ID	5049	11	9.73	< 0.001
Years of bison (YrsOB)	980	1	20.79	< 0.001
Number of burns in past 5 years (BP5Yrs)	322	1	6.82	0.010
Years since last burn (YrsSLB)	496	1	10.53	0.001
Residuals	9711	206		

*The abbreviated name for each parameter is given in parentheses

Table S5. ANOVA table (type II) for the RDA model of species composition in the Repeat analysis. The model explained 61% of variance in composition ($F_{33,206}$ = 9.78, p < 0.001). The statistics reflect the partial effect attributed to each variable after accounting for all other variables in the model.

**Explanatory Variables**	**Var**	**DF**	***F*-value**	***p*-value**
Site ID	13.65	19	12.80	< 0.001
Year ID	1.77	11	2.87	< 0.001
Years of bison	0.14	1	2.51	< 0.001
Number of burns in past 5 years	0.11	1	1.97	0.008
Years since last burn	0.10	1	1.80	0.012
Residuals	11.56	206		
